# Dysfunction of monocyte in the development of HBV-ACLF: Immune activation or immune suppression?

**DOI:** 10.1515/jtim-2025-0014

**Published:** 2025-07-30

**Authors:** Yangfan Shen, Xiaoxin Wu, Xiaowei Xu

**Affiliations:** State Key Laboratory for Diagnosis and Treatment of Infectious Diseases, National Clinical Research Centre for Infectious Diseases, Collaborative Innovation Centre for Diagnosis and Treatment of Infectious Diseases, The First Affiliated Hospital, Zhejiang University School of Medicine, Hangzhou, Zhejiang Province, China

## Introduction

HBV-related acute-on-chronic liver failure (HBV-ACLF) is a complex syndrome that develops in patients with chronic hepatitis B virus infection, characterized by acute liver failure and/or multi-organ failure, with a high risk of short-term mortality. Immune dysfunction is a key factor affecting the occurrence and progression of HBV-ACLF and recent studies have increasingly focused on the dysfunction of monocytes.^[1]^

Monocytes serve as the first line of defense against pathogen invasion in the host. It has been reported that patients with HBV-ACLF exhibit functional imbalances in the intrahepatic and peripheral monocytes.^[1,2]^ However, these research on monocyte has yielded contradictory results. Therefore, clarifying the precise dysfunction of monocytes and its underlying mechanisms may provide new therapeutic targets for immunotherapy in ACLF.

## Immune activation of monocyte in the development of HBV-ACLF

Patients with HBV-ACLF exhibit excessive and persistent hepatic and systemic inflammation, potentially driven by over-activated monocytes, which may exacerbate organ failure and increase mortality risk.^[1]^ In response to acute liver injury during the initiation of HBV-ACLF, the injured liver immediately releases various damage-associated molecular patterns (DAMPs), such as HMGB1, histones, IL-33, and inflammatory oxylipids, which interact with monocytes and/or macrophages via specific binding to pattern-recognition receptors (PRRs) expressed on the surface of monocytes, including TLR4 and ST2, resulting in immune activation^[3,4]^. Moreover, pathogens invading through the respiratory tract or a compromised intestinal barrier, the most common extrahepatic trigger for ACLF, can generate a variety of pathogen-associated molecular patterns (PAMPs), such as LPS, which also activate monocytes *via* PRRs.^[4]^ Although the functions of DAMPs and PAMPs are not identical, they collectively activate the monocytes and complicate the mechanism of ACLF.

Activated intrahepatic monocytes rapidly shift toward a pro-inflammatory phenotype, exhibiting elevated levels of pro-inflammatory markers such as CD80, CCR2, and the antigen-presenting molecule HLA-DR, along with an enhanced ability to release inflammatory cytokines such as TNF-α, IL-1β, and IL-6.^[3]^ These cytokines further activate peripheral monocytes, contributing to systemic inflammatory response syndrome (SIRS) and extrahepatic organ damage. Recent single-cell RNA sequencing studies have confirmed the transition of monocytes to a pro-inflammatory phenotype at the transcriptomic level in patients with ACLF and identified the upregulated expression of inflammatory-associated genes such as THBS1, SAMSN 1, and MALAT1, offering potential research targets for exploring the molecular mechanisms underlying this phenotype transition. Notably, stratified analyses has shown a more significant upregulation of pro-inflammatory genes such as IL-6, CCL4, CCL5, HES4, VIM, LGALS2, and TREM1 in monocytes collected from deceased ACLF patients, suggesting that the degree of monocyte immune activation may be positively correlated with poor prognosis in ACLF.^[5,6]^ In addition, activated monocytes can induce pyroptosis of iNKT cell, which futher impairs the immune function in ACLF patients.^[7]^

## Immune suppression of monocyte in the development of HBV-ACLF

Patients with end-stage ACLF exhibit a marked immunosuppressive state involving monocytes, which impairs host antimicrobial function, promotes secondary infections, and ultimately leads to adverse outcomes.^[8,9]^

It has been reported that liver-resident macrophages (Kupffer cells) are depleted during the development of HBV-ACLF, while TREM2+ monocytes are recruited into the liver from peripheral blood.^[10]^ These recruited intrahepatic monocytes undergo reprogramming into an immunosuppressive state, characterized by high expression of anti-inflammatory markers such as CD206 and IL-10, and low expression of pro-inflammatory genes like TNF-α and IL-1β.^[10]^ This may be a host response to repair damaged tissue and control excessive inflammation. Consistently, peripheral monocytes also revealed upregulation of immunosuppressive markers such as CD163 and MERTK, a reduction in the expression of HLA-DR, and impaired oxidative burst capacity.^[5,6,8,9]^ Recently, researchers identified a distinct subset of monocytes in ACLF patients, termed myeloid-derived suppressor cells (MDSCs), which display reduced pathogen-clearing capacity and suppress T cell proliferation and activation, thereby further impairing adaptive immune function.^[11]^

The transition to an immunosuppressive phenotype of monocytes is likely a result of prolonged stimulation by DAMPs, PAMPs, and cytokines, but the specific molecular mechanisms remain unclear.^[11]^ Recent studies in the metabolic field suggest that metabolic reprogramming may play a role in the immune suppression of monocytes, including amino acid metabolism imbalance mediated by the GLUL gene, lipid metabolism disorders induced by unsaturated fatty acids, and reduced tricarboxylic acid cycle metabolism.^[5,9,10]^ Futhermore, upregulation of inhibitory receptors PD-1 on monocytes has been observed in ACLF patients with sepsis, offering new insight into immune exhaustion for exploring the mechanisms phenotype transition in monocytes.^[12]^

## Conclusion

**Figure 1:**
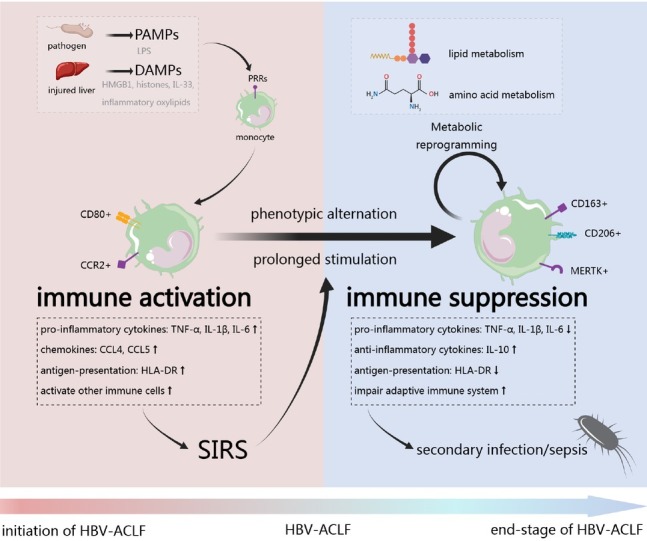
**The dysfunction of monocyte in the development of HBV-ACLF. PAMP: pathogen-associated molecular patterns; DAMP: damage-associated molecular patterns; HBV-ACLF: HBV-related acute-on-chronic liver failure; CCL: C-C motif chemokine ligand; TNF-**α**: tumor necrosis factor-alpha; IL: interleukin; HLA: human leukocyte antigen**.

These studies revealed that monocytes may play a dual role in the progression of HBV-ACLF. In the early stage, monocytes are rapidly activated into a pro-inflammatory state by exposed to DAMPs and PAMPs, leading to systemic inflammation and exacerbating multi-organ damage. Under prolonged stimulation by multiple pro-inflammatory factors, monocytes shift toward an immunosuppressive phenotype, which ultimately lead to secondary infections or sepsis. It is worth noting that both excessive immune activation and immune suppression are closely associated with poor prognosis in ACLF patients (Figure 1).

In summary, reshaping the immune homeostasis of monocytes may be a potential therapeutic target for ACLF. Given the considerable heterogeneity in severity and disease staging among HBV-ACLF patients, it is critical to investigate the evolutionary patterns of monocyte dysfunction and the underlying mechanisms, identify precise diagnostic and therapeutic targets, and provide an immunological foundation for the diagnosis and treatment of HBV-ACLF.
